# Characterization of the Particle Size Fraction associated with Heavy Metals in Suspended Sediments of the Yellow River

**DOI:** 10.3390/ijerph120606725

**Published:** 2015-06-15

**Authors:** Qingzhen Yao, Xiaojing Wang, Huimin Jian, Hongtao Chen, Zhigang Yu

**Affiliations:** Key Laboratory of Marine Chemistry Theory and Technology, Ministry of Education, Ocean University of China, Qingdao 266100, China; E-Mails: qzhyao@ouc.edu.cn (Q.Y.); wxj311@163.com (X.W.); jianhm@ouc.edu.cn (H.J.); chenht@ouc.edu.cn (H.C.)

**Keywords:** Yellow River, heavy metals, particle size, fluxes

## Abstract

Variations in the concentrations of particulate heavy metals and fluxes into the sea in the Yellow River were examined based on observational and measured data from January 2009 to December 2010. A custom-built water elutriation apparatus was used to separate suspended sediments into five size fractions. Clay and very fine silt is the dominant fraction in most of the suspended sediments, accounting for >40% of the samples. Cu, Pb, Zn, Cr, Fe and Mn are slightly affected by anthropogenic activities, while Cd is moderate affected. The concentrations of heavy metals increased with decrease in particle size. For suspended sediments in the Yellow River, on average 78%–82% of the total heavy metal loading accumulated in the <16 μm fraction. About 43% and 53% of heavy metal in 2009 and 2010 respectively, were readily transported to the Bohai Sea with “truly suspended” particles, which have potentially harmful effects on marine organisms.

## 1. Introduction

River transport is the principal pathway of suspended and dissolved elements from land to the sea. A wide range of heavy metals may be generated through human activities, then discharged into rivers by dynamic processes of soil erosion, through wastewaters, through industrial activities [1] and through agriculture [2]. Heavy metals may be brought by rivers to the coastal area, and then can be accumulated in marine sediments. Approximately 18.3 Gt of suspended sediment are transported to the ocean by rivers every year [3]. Large quantities of terrestrial sediments and their associated pollutants would have an important effect on coastal and marine environments [4,5,6,7,8,9].

The grain size is one of the main factors that govern heavy metal contamination in the particulate fraction. Generally, fine particles have a higher ability to carry the heavy metals due to the increase of specific surface area, and due to the presence of clay minerals, organic matter, and Fe/Mn/Al oxides associated forming fine-sized aggregates [10,11,12]. Thus, the impact of particle characteristics/physico-chemical properties on heavy metal concentration in the river-suspended particles should also be analyzed.

The Yellow River is known for its high sediment discharge with the averaged value of 1.6 × 10^9^ tons/yr [13], approximately an order of magnitude greater than the Yangtze River [14]. The upper reaches of the Yellow River drain supply about 60% of the river discharge but only 10% of the sediment load. This area mainly comprises sandstone, dolomitic limestone and minor volcanics [15]. The Loess Plateau, which covers part of the upper reaches and most of the middle reaches of the Yellow River comprises 3 × 10^5^ km^2^, or 40% of the total Yellow River drainage basin [16]. The Loess Plateau, while contributing 40% of the river discharge, with its easily erodible soils, contributes 90% of the Yellow River sediment load, which results in heavy metal concentrations in the Yellow River sediment that are similar to the values found in the Malan Loess [17]. Natural weathering processes in the Loess Plateau are of prime importance in controlling the particle heavy metal concentrations in the Yellow River [18]. The concentrations of major heavy metals in the Yellow River sediments are relatively lower than those of large rivers through industrialized regions [19,20,21]. Qiao *et al.* [18] demonstrated that the heavy metal concentrations in the Yellow River sediment transported to the sea have not changed significantly in the past 20–30 years. Despite these previous studies, few studies have focused on the concentrations of heavy metals in different particle size fractions. Moreover, since the fine particle fractions are often preferentially transported to offshore areas, they are potentially more harmful to the marine environment.

In order to assess the environmental risk and take appropriate measures in the future, it is important to know the size fractions in which the heavy metals are distributed. The objectives of this study are to: (1) assess the contamination levels of toxic Zn, Pb, Cu, Cr and Cd in suspended sediments in the Yellow River; (2) investigate the distribution patterns of heavy metals in different particle size fractions; (3) discuss the factors affecting heavy metals in different particle size fractions; and (4) evaluate the impact of particle size on heavy metal transport to the sea and its environmental effect.

## 2. Materials and Methods

### 2.1. Sample Collection

**Figure 1:**
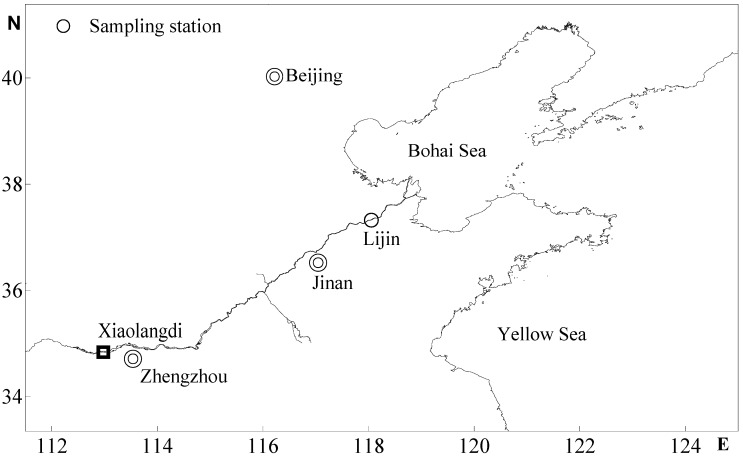
Locations of the Yellow River Basin and the sampling station.

Samples were collected from January 2009 to December 2010 at Station Lijin, which is located approximately 100 km upstream from the river mouth (Figure 1). A custom-built water elutriation apparatus, built according to Walling and Woodward [22], was used to separate suspended sediments into clay-very fine silt (<8 μm), fine silt (8–16 μm), medium silt (16–32 μm), coarse silt (32–63 μm) and sand (>63 μm). Approximately 100 to 150 L of surface water sample was collected. After allowing particulates to settle for approximately 24 h, the clear water was decanted and stored in a container for use as carrier water in the elutriation process, and the sediment that remained in the container was used for elutriation. When a sampling run was initiated, the water elutriator was filled with clear water that was just drawn from the container. After enough slurry was drawn into the apparatus, the clear water was drawn through the system until the sedimentation chambers were completely flushed. The sediment samples collected from each sedimentation chamber were filtered through 0.45 μm pore-size acid-washed Millipore filters and frozen. The slurry collected in the outflow containers was left to settle for approximately 72 h and was then decanted. The sediment was collected on filters and frozen.

### 2.2. Analytical Methods

For each particle size fraction and bulk suspended sediment, about 0.10 g of well-ground sample was digested using 5.00 mL HNO_3_, 2.00 mL HClO_4_ and 1.50 mL HF in a PTFE reactor at 160 °C for 6 h according to Li *et al.* [23]. The total amounts of Cu, Pb, Zn, Cr, Fe and Mn were determined by inductively coupled plasma-atomic emission spectrometry (ICP-AES, Thermo-6300). Cd was determined by graphite furnace atomic absorption spectrometry (GFAAS, Thermo-SOLLARM6). The detection limits were 0.016, 0.034, 0.005, 0.004, 0.0002, 0.014 and 0.002 mg·kg^−1^ for Cu, Pb, Zn, Cr, Cd, Fe and Mn, respectively. Reference standard of offshore Marine Sediment (GBW07314, The Second Institute of Oceanography SOA, Hangzhou, China) and reagent blanks were used as the quality control sample during the analysis procedures. The values obtained for all the studied elements fall in the required range of certified contents. The relative standard deviations (*n* = 3) of duplicate samples were less than 10% and 5%.

### 2.3. Assessment Methods

#### 2.3.1. Enrichment Factor (***EF**_x_*)

The enrichment factor was used to assess the anthropogenically introduced heavy metal, and it was calculated by Equation (1) [24]:

(1) $${EF}_{x}=X/X_{ref}$$

where ***X*** is the concentration in bulk suspended sediments (mg·kg^−1^) and ***X**_ref_* is the reference concentration (mg·kg^−1^). As we do not have heavy metal background values of our study area, we adopted the background concentrations of heavy metals in Chinese soils [25] from this study as the background value. The EF was classified as follows: >16.0 (excessive), 16–8.1 (very severe), 8.0–4.1 (severe), 4.0–2.1 (moderate) and 2.0–1.1 (slight) [26].

#### 2.3.2. Distribution Factor (***DF**_X_*)

In order to estimate which size fraction the heavy metals are preferentially enriched in, distribution factor (***DF**_X_*) was calculated by Equation (2) [27]:

(2) $${DF}_{X}=X_{fraction}/X_{bulk}$$

where ***X**_fraction_* and ***X**_bulk_* are the contents (mg·kg^−1^) of heavy metal in a given particle fraction and the bulk sediments sample, respectively. The heavy metal is assumed to be accumulated in this fraction, if ***DF**_X_* >1.

#### 2.3.3. Mass Loading

Another important index to assess the contamination of heavy metals is the mass loading. Loading combines heavy metal concentrations, on a grain size basis, with data on the mass percentage. The index was calculated by Equation (3) [28]:

(3) $${GSF}_{loading}=({HM}_{i}\times {GS}_{i})/(\sum_{i=1}^{5}({HM}_{i}\times {GS}_{i})\times 100)$$

where ***HM**_i_* is the heavy metal concentrations (mg·kg^−1^) in the individual grain size fraction (i) and ***GS**_i_* is the mass percentage of the individual fraction.

## 3. Result and Discussion

### 3.1. Particle Size Distribution of Suspended Sediments

**Figure 2:**
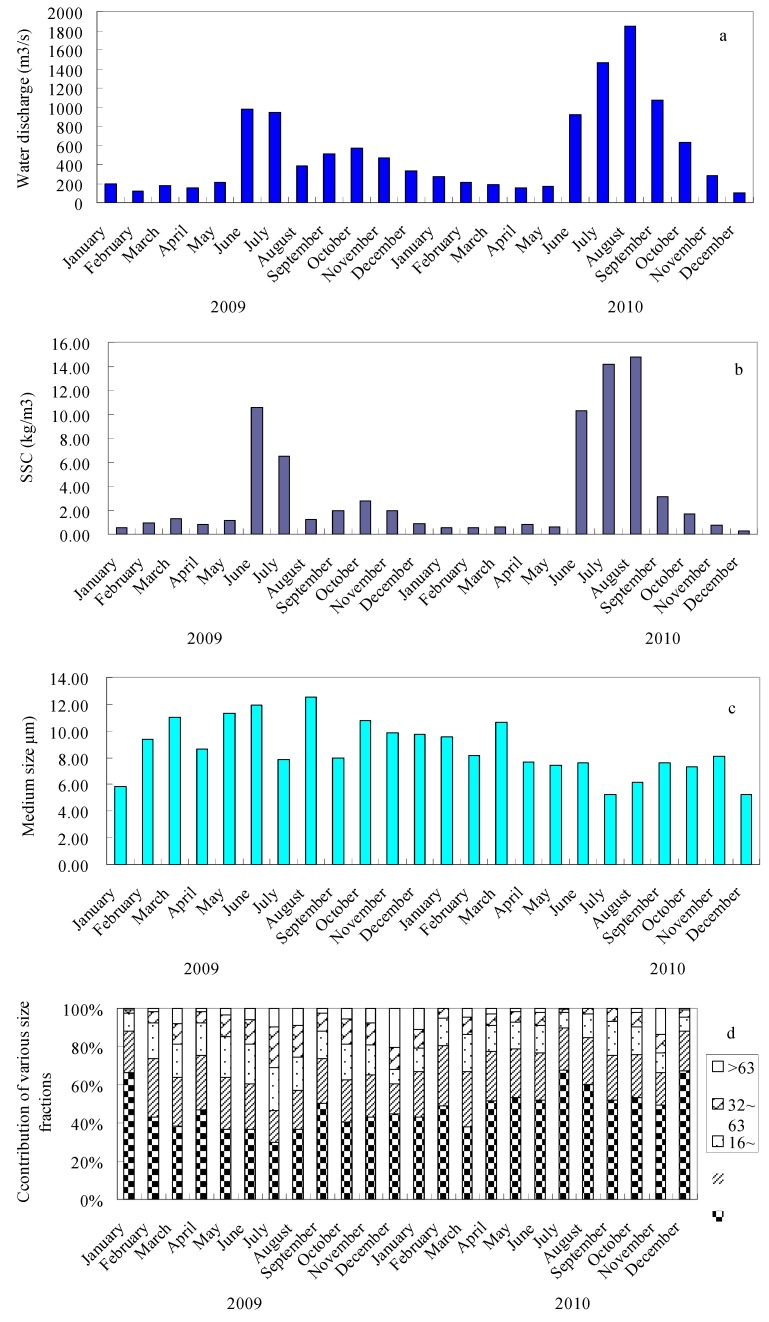
Water discharge (**a**), suspended sediment concentration (SSC) (**b**), medium size (**c**) and contribution of various size fractions to the suspended sediments (**d**) in the Yellow River.

The particle size distribution of suspended sediments in the Yellow River displays large variations. The contributions of various size fractions to the total suspended sediment of each sample are shown in Figure 2. The clay and very fine silt (<8 μm) of suspended sediments ranged from 36% to 67%, while the sand fraction (>63 μm) ranged from 0.9% to 20%. The contribution of the fine silt fractions (8–16 μm) ranged from 17% to 32%, whereas medium silt (16–32 μm) ranged from 10% to 30%, and coarse silt (32–63 μm) ranged from 7% to 24%. The increase in clay—very fine silt was accompanied by a decrease in medium silt, coarse silt, and sand fractions. It should be noted that clay and very fine silt is the dominant fraction in most of the suspended sediments, accounting for >40% of the samples. The fine silt fraction is generally less than 25%, medium silt fraction less than 15%, coarse silt fraction less than 10% and the sand fraction less than 5%.

### 3.2. Heavy Metals Variability and Enrichment

**Figure 3:**
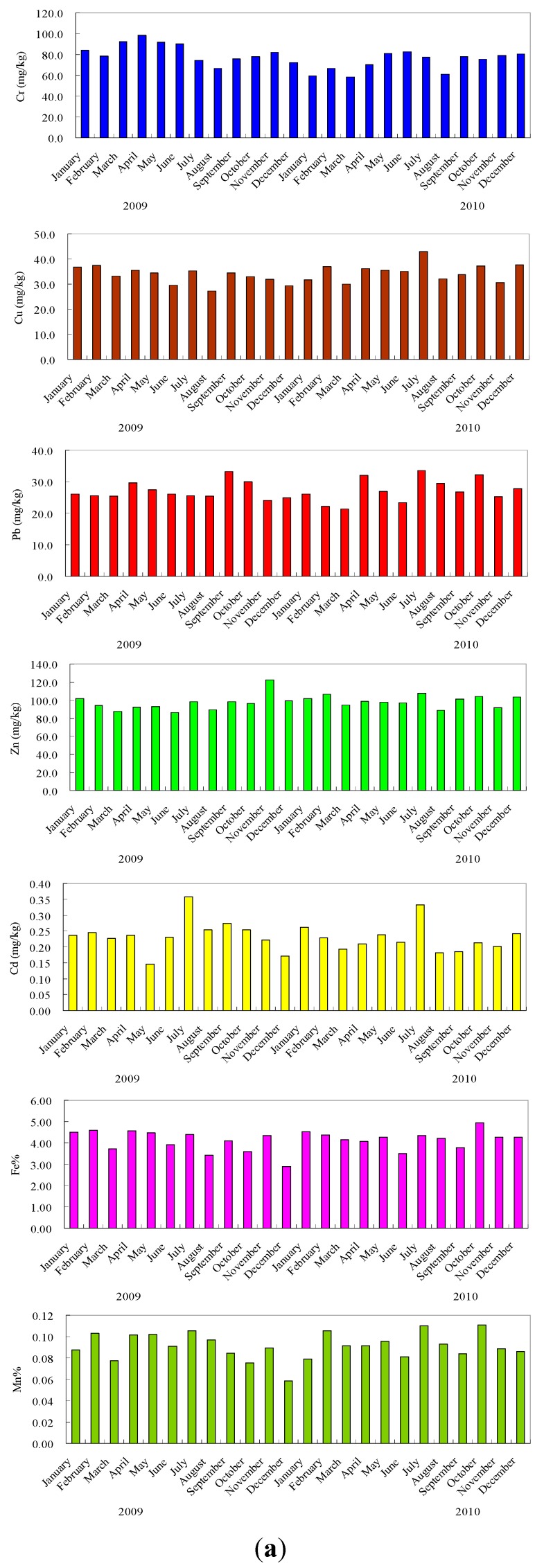

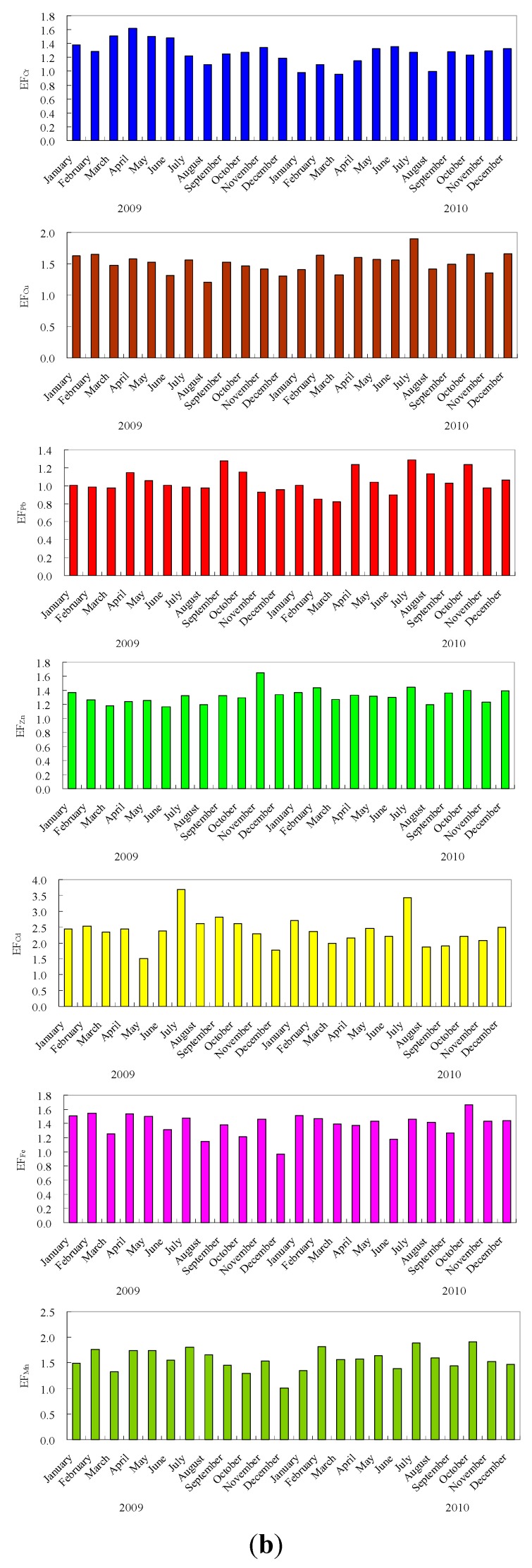
Heavy metal concentrations in suspended sediments and enrichment factors in the Yellow River.

The heavy metal contents in the suspended sediments decrease in the order of Fe > Mn > Zn > Cr > Cu > Pb > Cd. A statistical summary and other comparisons of the metal contents are presented in Table 1. From the table, the heavy metal contents varied greatly as follows: Cd, 0.15–0.36 mg/kg with an average of 0.23 mg/kg; Fe, 2.88%–4.95% with an average of 4.13%; Mn, 0.058%–0.111% with an average of 0.091%; Cr, 59–99 mg/kg with an average of 77 mg/kg; Cu, 27–43 mg/kg with an average of 34 mg/kg; Zn, 86–122 mg/kg with an average of 92 mg/kg; Pb, 21–34 mg/kg with an average of 27 mg/kg. The monthly change of heavy metal concentrations in the suspended sediments may largely result from the variation in the suspended sediments grain size (Figure 2). Heavy metals in the suspended sediments may originate from parent materials (particularly natural bedrock), industrial activities, traffic emissions, and municipal wastes [29]. The enrichment factor (EF) was used to assess the anthropogenically introduced heavy metal [30,31,32]. For bulk suspended sediments, the background concentration in Chinese soils [25] was used as the background in this study. If EF is greater than 1, the concentration of the element is higher than the background, and heavy metals may come from anthropogenic sources. The calculated values of EF for all the seven metals are shown in Table 1 and Figure 3. Average EF values for bulk suspended sediments are in order of Cd (2.39) > Mn (1.56) > Cu (1.51) > Fe(1.39) > Zn(1.31) > Cr (1.27) > Pb (1.04). By comparing the concentrations of the heavy metals in suspended sediments with those from previous studies of the Yellow River [18,19,21,33], the concentration of Cr was determined to be quite similar to that found in previous studies (Table 2). However, the concentrations of Pb and Zn were higher than those in previous studies [16,18,19,21], which indicates that the contribution of anthropogenic pollutants to the heavy metal concentrations in the suspended sediments has been increasing. A recent report [34] showed that the quantity of wastewater from the Yellow River drainage basin increased dramatically from ~2.0 Gt/yr during the 1980s to 4.36 Gt/yr in 2010. Thus, the increase in sewage discharge along the Yellow river drainage basin may result in heavy metal concentrations in suspended sediments increasing recently [19].

**Table 1:** Metal concentrations (in mg/kg except for Fe and Mn in %) in the TSS in the Yellow River.

Heavy Metal	Minimum	Maximum	Mean	Background Concentration in Chinese Soils ^a^
Cu	27(1.19)	43(1.90)	34	22.6
Pb	21(0.81)	34(1.31)	27	26
Zn	86(1.16)	122(1.64)	97	74.2
Cd	0.15(1.55)	0.36(3.71)	0.23	0.097
Cr	59(0.97)	99(1.62)	77	61
Mn	0.058(0.99)	0.111(1.90)	0.0912	0.0583
Fe	2.88(0.97)	4.95(1.67)	4.13	2.97

Notes: Data in parentheses are enrichment factor (EF). ${EF}_{x}=X/X_{ref}$ see definition in the text; ^**a**^ Wei *et al.*, [32].

**Table 2:** Comparisons of the heavy metal concentrations in the suspended sediments with those of previous studies in the Yellow River (mg/kg).

Sample Time	Cu	Pb	Zn	Cr	Mn	Data Sources
1980s	26.7	16.4	69.8	76.9	767	[16,19]
1997–1998	17.6	29.5	60.2	65.4	431.1	[33]
Nov. 2000	22.99	19.38	76.78		600	[18]
Aug. 2001	10.31	10.82	70.03		500	[18]
Jun. 2009		24.0	63.1	44.7	459	[21]
2009–2010	34.0	27.0	97.0	77.0	912	This study

Fe and Mn are the most abundant metals in all suspended sediments because these metals are common elements in the Earth’s crust [35]. The maximum and minimum concentration of Fe and Mn is observed in October 2010 and in December 2009, respectively. EF_Fe_ and EF_Mn_ varied from 0.97 to 1.67 and from 0.99 to 1.90 with an average of 1.39 and 1.56, respectively. From this comparison, concentrations of Fe and Mn are slightly affected by anthropogenic activities.

The main anthropogenic sources of cadmium relate to metallurgical industries, municipal effluents, sewage sludge and mine wastes. Other sources are fossil fuels and some phosphorus-containing fertilizers [36]. The maximum (0.36 mg/kg) and minimum (0.15 mg/kg) concentrations of Cd are observed in July and May 2009 respectively. The EF_Cd_ were almost above 2.0 except that in May (1.51), December (1.77) 2009 and August (1.81) and September (1.91) 2010. The Yellow River drainage basin covers an area of 7.95 × 10^5^ km^2^, in which the area of farmland is 1.19 × 10^5^ km^2^, approximately 15% of total land area [37]. These are the source of Cd in the present suspended sediments. The Cd in Yellow River-suspended sediments may be accumulated from agricultural lands.

Chromium is an essential trace element required for the metabolism of lipids and proteins. Normally, Cr exists in two possible oxidation states in soils and sediments: The trivalent Cr(III) and hexavalent Cr(VI) which may originate either from weathering of chromites or from industrial activities. Hexavalent chromium being mobile and extremely toxic, which is more harmful than trivalent Cr [38]. In the present study, the maximum (99 mg/kg) and minimum (55 mg/kg) concentration of Cr were observed in April 2009 and January 2010 respectively. Kulahci and Sen [39] pointed out that more than 70% of Cr in the environment comes from the man-made emissions, primarily from metal use. EF_Cr_ varied from 0.95 to 1.61 in the Yellow River, with an average of 1.27. Thus, the higher concentration of Cr in the Yellow River may be due to anthropogenic inputs.

Copper can be retained by sediment through exchange and specific adsorption mechanisms but precipitation may also be an important mechanism for retention in polluted sediments. Cu can easily complex with organic matters because it is easy to form high stability constants of organic-Cu compounds [40,41]. This is significantly reflected in the distribution of organic matter in the polluted sites of the river sediments. Agrochemicals (especially phosphorite fertilizers) and residential waste are the major sources of the Cu in the river sediments [41,42]. In the present study, the maximum (43 mg/kg) and minimum (27 mg/kg) concentrations of Cu were observed in July 2010 and August 2009 respectively. The high concentration of copper in the Yellow River may be due to the high content of organic matter along with residential wastes and also chemicals.

Lead is one of the potentially hazardous elements in the sediments and it is the least mobile element among the toxic elements. Pb impedes the synthesis of hemoglobin and accumulates within red cells as well as bones to give rise to anemia, headache and dizziness [38]. Pb is one of the most important emissions from vehicles, which may cause air, water and soil pollution [43]. In the present study, the concentration of Pb was higher (34 mg/kg) in July 2010 and low (21 mg/kg) in March 2010 (Figure 3). Most of EF_Pb_ in the suspended sediments are around 1.0. Thus, the concentration of Pb is not obviously affected by anthropogenic activities.

Sediments are the primarily sink for Zn. It is preferentially associated with fine grained particles or is absorbed by the clay minerals [44]. Zinc belongs to a group of trace metals that are potentially most dangerous for the biosphere. Krishna and Govil [38] pointed out that higher concentrations of Zn cause hematological disorders. The main sources of Zn are from industries and the use of liquid manure and composted materials. The maximum (122 mg/kg) and the minimum (86 mg/kg) concentrations of Zn were observed in November and June 2009, respectively. Most of EF_Pb_ in the suspended sediments are <1.5. Thus, the concentrations of Zn are slightly affected by anthropogenic activities.

### 3.3. Distribution of Metals in Particle Size Fractions

**Figure 4:**
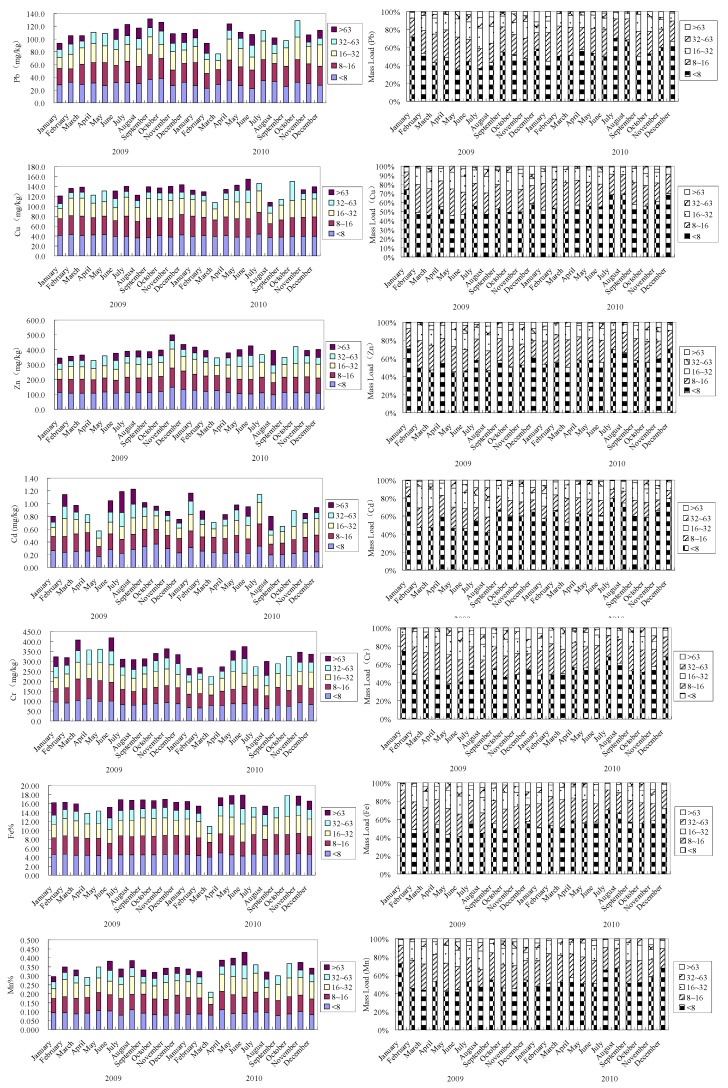
Relative contribution of heavy metal to that in bulk sediments and mass loading of five sediments particle size.

**Figure 5:**
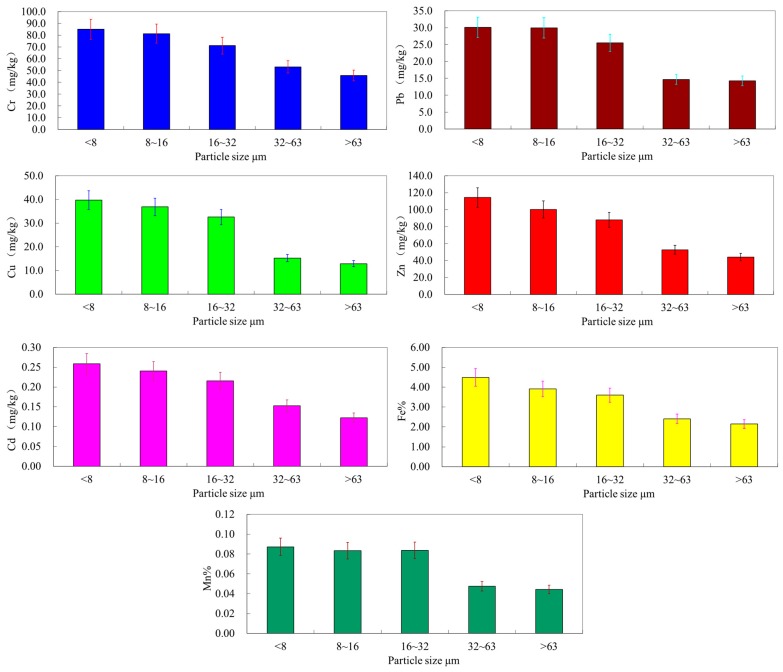
Heavy metal concentrations in different size suspended particles.

The concentrations of heavy metals (Cu, Zn, Pb, Cd, Cr, Mn, Fe) in the particle size fractions are shown in Table 3, Figure 4 and Figure 5. It was obvious that heavy metals were not homogeneously distributed among the various particle fractions, suggesting the influence of sediment texture on the partitioning of heavy metals in the suspended sediments. In general, the concentrations of all heavy metals in the clay and fine silt (<8 μm), and the fine silt fractions (8–16 μm) were higher than in the bulk suspended sediments. Heavy metals tended to accumulate in the fine fractions, which can be indicated more clearly by the distribution factors (DFs) (Table 3). The concentrations and DFs of heavy metals in different size classes demonstrated higher accumulation in finer fractions, and the trace metals mainly accumulated in the fractions of clay and fine silt (Figure 5). Furthermore, metal accumulation in these two fine fractions was especially noticeable for Cu (1.18, 1.09), followed by Zn (1.16, 1.02) > Pb (1.15, 1.15) > Fe (1.14, 1.02) > Cd (1.13, 1.04) > Mn (1.12, 1.11) > Cr (1.10, 1.05) (Figure 3). However, the concentrations and DFs of trace metals in the coarse silt (32–63 μm) and sand (>63 μm) were generally low.

**Figure 6:**
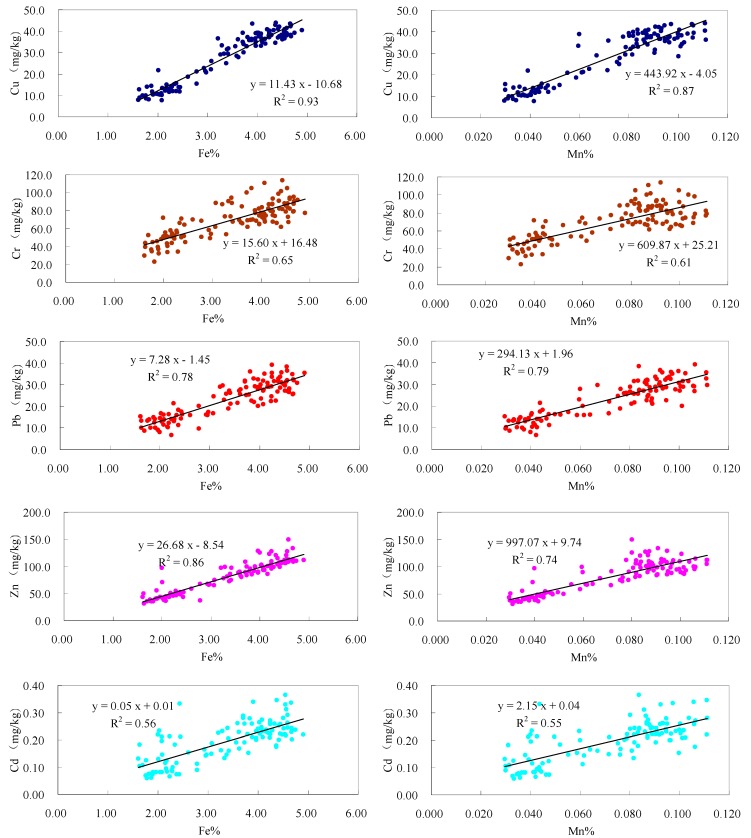
The correlations between heavy metals and the contents of Fe as well as Mn in different size fractions.

Our data were in line with previous reports on the preferential partitioning of trace metals to fine soil particle size fractions, especially the maximum accumulation of some heavy metals in the clay fraction [45,46,47,48]. These might be attributed to the great surface area per unit of mass of the fine particles, which increases the adsorption capacity of these fractions. Furthermore, finer suspended sediment particles may accumulate higher concentrations of heavy metals due to the high content of secondary minerals (clay minerals, Fe, Mn, Al oxides and hydroxides, and carbonates) and organic matter [49]. Sand and silt fractions in sediments are largely composed of the primary mineral quartz (e.g., SiO_2_), which is a very weak adsorbent for heavy metals. The statistical results of the correlations analysis between metals and the contents of Fe as well as Mn in different fractions of the samples (Figure 6) further support the potential sources and behavior of the heavy metals.

**Table 3:** Metal concentrations (in mg/kg except for Fe and Mn in %) in the suspended sediments of different particle size in the Yellow River.

Heavy Metal		<8 µm	8–16 µm	16–32 µm	32–63 µm	>63 µm
Cu	Concentration	36–44	32–43	20–39	10–30	8-22
	Mean	40 (1.18)	37 (1.09)	33 (0.97)	15 (0.44)	11 (0.32)
Pb	Concentration	22–38	21–39	14–35	10–25	9–17
	Mean	30 (1.15)	30(1.15)	25 (0.96)	16 (0.62)	13 (0.50)
Zn	Concentration	98–150	80–127	66–126	37–73	31–65
	Mean	114(1.16)	100 (1.02)	88 (0.90)	52 (0.53)	41 (0.42)
Cd	Concentration	0.17–0.36	0.15–0.35	0.13–0.34	0.07–0.24	0.06–0.22
	Mean	0.26 (1.13)	0.24 (1.04)	0.21 (0.91)	0.13 (0.57)	0.13 (0.57)
Cr	Concentration	62–114	54–111	51–90	32–95	23–72
	Mean	85 (1.10)	81 (1.05)	71 (0.92)	53 (0.69)	45 (0.58)
Mn	Concentration	0.078–0.111	0.076–0.111	0.039–0.106	0.030–0.082	0.030–0.072
	Mean	0.092 (1.12)	0.091 (1.11)	0.084 (1.02)	0.048 (0.059)	0.038 (0.46)
Fe	Concentration	3.71–4.90	3.21–4.48	2.01–4.06	1.63–3.37	1.60–3.07
	Mean	4.49 (1.14)	4.03 (1.02)	3.06 (0.77)	2.41 (0.61)	2.02 (0.51)

The effects of clay minerals and oxides might be demonstrated by the accumulation of Fe and Mn in the fine fractions (Figure 6), and the significantly positive correlation coefficients of heavy metals with them in all suspended sediment particle fractions (Figure 6). The correlation coefficients of Cu, Pb, and Zn with Fe were much higher than those of Cd, and Cr with Fe, and the correlation coefficients of Cu, Pb, and Zn with Mn were also relatively higher than those of Cd, and Cr with Mn. In general, the distribution of heavy metals within different particle size fractions might be a function of mineral composition and the amount of adsorption sites in each particle fraction.

The proportions of metal loadings in different size fractions compared to the total in the suspended sediments were calculated from the metal concentration and the mass percentage of each particle fraction (Figure 4). The results showed that heavy metal loadings (%) were also different among various particle fractions of the suspended sediments. Similarly, finer particle fractions usually showed proportionally higher heavy metal loadings than the mass fraction they represent. For suspended sediments in the Yellow River, on average 78%–82% of the total heavy metal loading accumulated in the <16 μm fractions (Figure 4), and 53%–54% in the <8 μm fractions, both higher than the corresponding suspended sediments mass percentage (71% of <16 μm, 48% of <8 μm).

### 3.4. Importance of Particle Size Fractions in the Transport of Heavy Metal to the Bohai Sea

The suspended sediment load was 0.25 × 10^8^ tons/yr in 2009 and 1.25 × 10^8^ tons/yr in 2010. According to the monthly concentrations of heavy metals in the suspended sediments and the monthly suspended sediments loads in 2009 and 2010, the Cu, Pb, Zn, Cr and Cd fluxes were 1792, 1485, 5212, 4655 and 15 tons/yr in 2009, and 4557, 3631, 12,281, 9139 and 29 tons/yr in 2010 (Table 4). The fluxes in 2010 were approximately twice as high as those in 2009 due to the higher suspended sediment load.

**Table 4:** Annual fluxes of heavy metal in the Yellow River (metric ton/yr).

Heavy Metal		<8µm	8~16µm	16~32µm	32~63µm	>63µm	Total
Cu	2009	949	434	236	113	60	1792
	2010	2414	1104	601	287	151	4557
Pb	2009	787	360	196	94	49	1486
	2010	1923	880	479	229	120	3631
Zn	2009	2761	1263	687	329	173	5213
	2010	6505	2976	1620	774	407	12282
Cd	2009	8.0	3.7	2.0	1.0	0.4	15.1
	2010	15.8	7.2	3.9	1.9	1.0	29.8
Cr	2009	2466	1128	614	293	154	4655
	2010	4841	2215	1205	576	302	9139

When the velocity of water flow is lower, most river-suspended sediments are deposited in the estuary, and due to differences in settling velocities, the different particle sizes of the suspended sediment are deposited in different parts of the estuary [50]. The coarse suspended sediments (such as sand and coarse silt) are deposited closer to the land, whereas fine suspended sediments (such as fine silt) are transported a greater distance seaward, and the finest suspended sediments (such as clay) are delivered to the sea. Alber [51] operationally separated suspended sediments into “truly suspended” and “settleable” fractions with a cut-off velocity of 0.006 m/s and found that all measured parameters (Chl-a, organic carbon and nitrogen) were largely associated with the “truly suspended” fraction. Alber [51] hypothesized that the more organic-rich, biologically active material associated with the suspended fractions likely had a different fate in the estuary because “truly suspended” particles will be readily transported, but “settleable” particles will settle and be resuspended with each tide. According to the diameters of the elutriator chambers and the flow rate, the settling velocities of particles with size ranges of <8 μm, 8–16 μm, 16–32 μm, 32–63 μm and >63 μm were <0.004 cm/s, 0.004–0.016 cm/s, 0.016–0.064 cm/s, 0.064–0.256 cm/s and >0.256 cm/s, respectively. Thus, the <8 μm particle fraction could be regarded as “truly suspended” particles.

Based on the heavy metal concentrations in various particle size classes, the suspended sediment size distribution and the suspended sediment load, the riverine fluxes of particulate heavy metal to the Bohai Sea could be calculated in terms of total suspended sediment and various particle size classes. The results are shown in Table 4. Approximately 949, 787, 2761, 8.0, 2466 tons/yr of Cu, Pb, Zn, Cd, Cr in 2009 and 2414, 1923, 6505, 15.8, 4841 tons/yr of Cu, Pb, Zn, Cd, Cr in 2010 were associated with the “truly suspended” fraction (Table 4). In other words, 43% and 53% of heavy metal in 2009 and 2010 were readily transported to the Bohai Sea. Heavy metal would be released from the riverine sediments after contact with seawater [52,53,54,55]. The metal release resulted from the balance between two processes: (1) metal mobilization due to ionic exchange or degradation of organic complexes and (2) metal re-adsorption on solid phase [52], both processes depending on the nature of the solids, the particles size, the pH of the solution, and the metal [56,57]. Oursel *et al.* [55] reported that Cu, Co, Ni and Zn exhibited similar release patterns, with a maximal release between 4% and 30%, whereas Cd exhibited a higher release, between 11% and 100%, and Pb a release lower than 1.1%. Thus, the transfer of fine particles to the open sea is probably accompanied by a non-negligible pollutant release to the dissolved compartment, which have potentially harmful effects on marine organisms.

## 4. Conclusions

The particle size of suspended sediment in the Yellow River displays large variations. Clay and very fine silt is the dominant fraction in most of the suspended sediments, accounting for >40% of the samples. The suspended sediments in the Yellow River have been slightly polluted by Cu, Pb, Zn, Cr, Fe and Mn, while Cd is moderately affected by anthropogenic activities. The heavy metal concentrations in the Yellow River were much higher than those reported in previous studies because of increasing pollution in the sediment of the river basin caused by human activity. The concentrations of heavy metals in particle fractions of suspended sediments generally increase as particle size decreases. The strong association between Fe, Mn and heavy metals can be explained by the role of particle size and Fe/Mn oxides in controlling metal concentrations. The loadings of heavy metals exhibited substantial accumulation in particle size fractions <32 μm, especially in the finest fractions (<16 μm). About 43%–53% of heavy metals were readily transported to the Bohai Sea with “truly suspended” particles, which may have potentially harmful effects on marine organisms.
